# Determining the nutritional values of new corn varieties on pigs and broilers

**DOI:** 10.3389/fvets.2024.1358773

**Published:** 2024-02-08

**Authors:** Hongrui Cao, Lei Yan, Xiaoming Song, Ling Liu, Shaohua Xu, Shengdi Hu, Shuai Zhang

**Affiliations:** ^1^State Key Laboratory of Animal Nutrition and Feeding, College of Animal Science and Technology, China Agricultural University, Beijing, China; ^2^New Hope Liuhe Co., Ltd., Chengdu, China

**Keywords:** new corn variety, nutritional value, growing pig, broiler, iron, cadmium, phytate phosphorus

## Abstract

The objective of this study was to evaluate the nutritional values of three new corn varieties (high-iron corn, cadmium-resistant corn, low-phytate phosphorus corn) cultivated with molecular marker-assisted selection breeding technique fed to growing pigs and broilers. Exp. 1 was conducted to compare the nutritional values of high-iron corn, high-chromium corn, low-phytate phosphorus corn and conventional corn fed to growing pigs based on a 15 × 2 Youden square design. Exp.2 was conducted to compare the nutritional values of high-iron corn, low-phytate phosphorus corn and conventional corn fed to broilers based on a completely randomized design. Parameters including nutrient digestibility, available energy and amino acids, and mineral deposition were measured. The results shows that the iron content in the high-iron corn and the cadmium content in the cadmium-resistant corn were 29.608 mg/kg and 0.0057 mg/kg, respectively, both were greater than those in the other three kinds of corns. When fed to growing pigs, the neutral detergent fiber digestibility of the high-iron corn group was lower than that of the conventional corn group (*p* < 0.05), and the acid detergent fiber digestibility of the high-iron group and the low-phytate phosphorus corn group was lower than that of the conventional corn group (*p* < 0.01). In addition, the digestible energy value of the high-iron corn in growing pigs was lower than that of the conventional corn (*p* < 0.05). When fed to broilers, it was observed that the tibia length of the low-phytate phosphorus corn group and the high-iron corn group was lower than that of the conventional corn group (*p* < 0.05). Moreover, the iron emission in feces of broilers fed the low-phytate phosphorus corn was lower than those fed the conventional corn and the high-iron corn (*p* < 0.05). In conclusion, modern breeding techniques could provide new plant ingredients which have potential benefits to pig and broiler production, but the comprehensive effects may be better when applied to growing pigs considering growth performance and environment effects. The breeding techniques related to the current study rarely changed the available energy values of the corn in growing pigs and broilers.

## 1 Introduction

Corn is indigenous to South America, with current distribution predominantly found in North and Central America. It ranks among the three highest-yielding cereal crops globally, and is a multipurpose crop, which can serve as a source of animal feed, fuel ethanol, food, and other industrial materials. China is among the greatest global producers of corn, with cultivation on an area of 41.26 million hectares and an estimated production of 260.67 million tons in 2021 (1). In China, corn has greater prominence as feed, thereby impacting the production of animal husbandry as an energy source. In 2020, U.S. corn exports reached $9.2 billion, an increase of $1.6 billion (20%) over the same period last year, and China’s strong feed demand contributed to this sharp increase (2).

The digestible energy (DE) value of corn on growing pigs is 3,451 kcal/kg (3) and the metabolizable energy (ME) value on poultry is 3,084 kcal/kg (4). The high available energy value of corn is mainly contributed to its high content of starch (can reach 72%) (5), and the low content of soluble non-starch polysaccharides (6). The crude protein (CP) content in corn is relatively low, ranging from 7 to 9% (7), and the essential amino acids contents are also low, containing only 0.25% lysine and 0.07% tryptophan (8). However, the fat content in corn is relatively high, about 3 to 4%, and is rich in oleic acid and linoleic acid, which is approximately 27 and 59%, respectively (2). In terms of mineral content, corn is low in calcium (Ca, only 0.01%) (8) and high in phosphorus (P, approximately 0.28%), of which 68% is phytate phosphorus (9). Among the trace elements, the levels of iron (Fe), copper (Cu), manganese (Mn), zinc (Zn), and selenium (Se) are all low. Phosphorus is required for diverse biological processes in animals, such as the skeletal system, and the cell membrane structure, but is primarily stored in the form of phytates in plant seeds, thus being poorly available for monogastric livestock including pigs and poultry. In addition, phytate has the capacity to chelate positively charged cations, especially calcium, iron and zinc, and can probably compromise the utilization of other dietary nutrients, including protein, starch and lipids (10). Iron also plays important role in animal health because it is a vital component of the hemoglobin in the erythrocyte. Thus, decreasing the phytate phosphorus content or increasing the iron content in plant seeds through breeding techniques can greatly benefit the utilization of those plant materials in animal feed. On the contrary, cadmium (Cd), as a nonessential trace transition metal, is a carcinogen and a possible mutagen to animals and humans. The high-cadmium pollution in soil also inhibits the normal growth and production of plants. Production of high-cadmium resistant breeds through breeding techniques can greatly take advantages of those polluted soil resources. But it is still under exploration of how to use those plant materials in the downstream production.

Therefore, the objective of this study was to evaluate the nutritional values of three new corn varieties (high-iron corn, cadmium-resistant corn, low-phytate phosphorus corn) cultivated with molecular marker-assisted selection technique fed to growing pigs and broilers.

## 2 Materials and methods

The three new corn varieties used in this study were developed with molecular marker-assisted selection breeding technique using the Complete-diallel design plus Unbalanced Breeding-like Inter-Cross (CICUB) population developed by Prof. Jianbing Yan from Huazhong Agricultural University (11). All corn samples were cultured and provided by Prof. Daiyin Chao from Chinese Academy of Science Center for Excellence in Molecular Plant Sciences.

### 2.1 Exp.1: chemical compositions, available energy, and amino acid digestibility in three new corn varieties fed to growing pigs

The animal trial was carried out in Fengning Animal Experiment Base of China Agricultural University (Hebei, China). Fifteen healthy barrows (Duroc × Landrace × Yorkshire) with an initial body weight of 29.73 ± 1.73 kg, fitted with T-shaped cannula were selected. After two weeks of recovery from surgery, all pigs were allotted to 5 experimental diets based on a 15 × 2 Youden square design. Specifically, the 5 experimental diets included 1 diet with the conventional corn, 3 test diets with the three new corn varieties, and 1 nitrogen-free diet. The animal trial contained 2 phases, each phase with 14 days, including 5 days for diet adaptation, 3 days for feces and urine collection, 2 days for digesta collection, 3 days for total heat production measurement in respiration calorimeter (chambers), and 1 day for fasting heat production measurement in respiration chamber. In each phase, the 5 experimental diets were randomly allotted to 15 pigs with 3 pigs per replicate. The compositions of the experimental diets are shown in Table 1. While commercial diets were provided for pigs for feeding *ad libitum* after surgery, the experimental diets were provided as an amount of 4% of the pig’s initial body weight in powder form. The daily feeding was divided into two equal meals and supplied twice at 0830 and 1,630. All pigs had free access to water via duckbill waterers. To ensure accurate assessment of feed intake, all scattered and leftover feeds were collected, dried, and weighed during the trial.

**Table 1:** Ingredient compositions of the experimental diets used in Experiment 1 fed to growing pigs (as-fed basis, %).

Ingredients	Corn diet	Nitrogen-free diet
Corn	99.7	-
Chromium trioxide	0.3	0.3
Corn starch	-	77.7
Soybean oil	-	3.0
Sucrose	-	15.0
Cellulose acetate	-	4.0
Total	100.0	100.0

Feces and urine samples from all pigs were promptly collected and were stored in −20°C until the end of the collection period, followed those described by Song et al. (12) and Ren et al. (13). The collected feces for 3 days were mixed and weighed, and the initial moisture content was measured. Subsequently, 20% of the total fecal collection was dried at 65°C for 72 h, kept at room temperature for 24 h, weighed, and then stored as a fecal sample at −20°C for further analysis. Urine was collected once daily at 1400, and 10 mL of 6 mol/L hydrochloric acid was added to the collection bucket to maintain a pH below 5.5 to prevent nitrogen loss by ammonia volatilization. The collected urine was passed through 4 layers of gauze, and a fixed proportion of 1% of the filtered urine was collected into bottles, then immediately stored at −20°C for further analysis.

The digesta of each pig was collected on days 9–10 followed the method developed by Adeola (14). A sample bag was placed at the outer end of the cannula after feeding at 0830, and digesta were collected continuously for 8 h until 1,630 every day. To minimize the impact of collection on the wound, the sample bag was changed when it was filled to a capacity, and then immediately stored at −20°C to prevent microbial fermentation in the collected digesta. After the collection period, all the digesta collected in two days were thawed naturally and mixed, and 500 mL subsample from each pig was freeze-dried using freeze-drier (Dongfulong Freeze-Drying System, Shanghai, China), and then went through 60-mesh filter and were bagged for further analysis.

On days 11–14 of each experimental phase, pigs were transferred to open-circuit respiration chambers for calorimetry test. The chambers were designed and built specially for pigs by China Agricultural University. Pigs in the respiration chambers were fasted on day 14. To avoid the influence of heat production from activity, data in respiration chambers used for fasting heat production calculation were collected from 2,230 on day 14 (about 31 h of fasting) to 0630 on day 15. The data of oxygen consumption and carbon dioxide and methane production for the 8 h were extrapolated to 24 h to estimate the daily maintenance energy. All pigs were weighed before entering the respiration chambers on day 11, before the fasting stage on day 14, and at the end of the fasting stage on day 15 in each phase.

The dry matter (DM), CP, ether extract (EE), ash and neutral detergent fiber (NDF) contents in samples of corn and diets were determined followed the procedures of GB/T 6435–2014 (15), GB/T 6432–2018 (16), GB/T 6433–2006 (17), GB/T 6438–2007 (18) and GB/T 20806–2006 (19), respectively. Total starch content in corn and diets was determined followed the procedures in AOAC (procedure 979.10; AOAC 2006) (20). Acid detergent fiber (ADF) content in corn and diets was determined followed the procedures of NY/T 1459–2007 (21). The gross energy (GE) content in corn, diets, feces and urine samples was measured using an oxygen bomb calorimeter (Model 6,400, Parr Corporation, USA) followed the ISO 9831:1998 standard (22). The contents of 15 amino acids in corn, diets and feces were determined followed the procedures of GB/T 18246–2000 (23), with the samples being hydrolyzed with 6 mol/L HCl at 110°C for 24 h and then determined using an automatic amino acid analyzer (Hitachi L-8900, Hitachi Group, Japan). The determination of sulfur-containing amino acids followed the procedures of GB/T 15399–2018 (24), with the samples being oxidized by performic acid at 0°C for 16 h, hydrolyzed by HCl for 24 h, and then determined using an automatic amino acid analyzer (Hitachi L-8800, Hitachi Group, Japan). The determination of tryptophan followed the procedures in Appendix A of GB/T 18246–2000 (23), with the samples being hydrolyzed at 110°C for 22 h with 4 mol/L sodium hydroxide, and then determined using a high-performance liquid chromatograph (Agilent 1,200, Agilent Technologies, Inc., USA). The determination of Ca, Cu, Fe, Zn and Mn content in corns using atomic absorption spectrophotometer followed the procedures of GB/T 13885–2017 (25). The determination of total P content followed the procedures of GB/T 6437–2018 (26). The determination of Cd content followed the procedures of GB/T 13082–1991 (27).

The major formula used for calculation in Exp. 1 was as follows:

(1) The exchanged gas volume in the standard state (0°C, 1,013 hPa):
$$SV=V\times \frac{P-Pw}{1013}\times \frac{273}{273+T}$$

$$V=time\left(\min\right)\times \mathrm{gas}\;\mathrm{flow}\;\mathrm{rate}\left(L/\min\right)$$

$$P_{w}=\frac{RH}{100}\times \left(3.999+0.45547T+0.001708T^{2}+0.000469T^{3}\right)$$

where SV is the standard volume of the exhaust gas (0°C, 1,013 hPa); V is the actual volume of the exhaust gas; P is the air pressure in the respiration chamber; P_W_ is the water vapor pressure; T is the temperature in the respiration chamber; RH is the relative humidity in the respiration chamber.

(2) The oxygen consumption and carbon dioxide production of each animal:
$$\begin{array}{l} V_{co_{2},\mathrm{production}}=\left(C_{co_{2},b}-C_{co_{2},a}\right)\times SV_{\mathrm{chamber}}+\left(C_{co_{2},a}-C_{co_{2},\mathrm{air}}\right)\\ \;\times SV_{\mathrm{exhaust}} \end{array}$$

$$\begin{array}{c} V_{o_{2},\mathrm{consumption}}=\left(C_{o_{2},a}-C_{o_{2},b}\right)\times SV_{\mathrm{chamber}}\\ +\;\left(C_{o_{2},\mathrm{air}}-C_{o_{2},a}\right)\times SV_{\mathrm{exhaust}} \end{array}$$

where C_CO2, a_ and C_CO2, b_ are the concentrations of carbon dioxide at the beginning and the end of respiration, %; C_O2, a_ and C_O2, b_ are the concentrations of oxygen at the beginning and the end of respiration, %; SV_chamber_ is the standard volume of the net used gas in the chamber; SV _exhaust_ is the standard volume of the exhaust gas.

(3) The heat production of each animal (28):
$$\begin{array}{c} \mathrm{Heat}\;\mathrm{production}\left(\mathrm{kJ}\right)=16.1753\times O_{2}\left(L\right)+5.0208\times CO_{2}\left(L\right)\\ -\;2.1673\times CH_{4}\left(L\right)-5.9873\\ \times \;N\left(\mathrm{urine}\;\mathrm{nitrogen},g\right) \end{array}$$
(4) The apparent total tract digestibility (ATTD) of nutrients in diets and corns^14^:
$$\mathrm{ATTD}\;\mathrm{of}\;\mathrm{dietary}\;\mathrm{nutrients}\left(\%\right)=\frac{\begin{array}{l} \mathrm{the}\;\mathrm{amount}\;\mathrm{of}\;\mathrm{nutrients}\;\mathrm{intake}\\ -\;\mathrm{the}\;\mathrm{amount}\;\mathrm{of}\;\mathrm{nutrients}\;\mathrm{in}\;\mathrm{feces} \end{array}}{\mathrm{the}\;\mathrm{amount}\;\mathrm{nutrients}\;\mathrm{intake}}\times 100$$
(5) The DE, ME and net energy (NE) values of corn:
$$DE_{d}=\left(GE-FE\right)/FI$$

$$DE_{c}=DE_{d}/0.997$$

$$ME_{c}=ME_{d}/0.997$$

$$NE_{d}=\left(ME_{d}-HP+FHP\right)/FI$$

$$NE_{c}=NE_{d}/0.997$$

where DE_d_, ME_d_ and NE_d_ are the DE, ME and NE values in each test diet (MJ/kg of DM); GE is the total GE intake of each pig (MJ of DM); FI is the actual feed intake during the collection period; FE and UE are the GE contents in feces and urine of each pig (MJ/kg of DM) over the collection period; HP is the total heat production of each pig over the collection period; FHP is the fasting heat production of each pig over the collection period; DE_c_, ME_c_ and NE_c_ are the DE, ME and NE values of each new corn variety (MJ/kg of DM) and 0.997 is the percentage of corn included in the test diet.

(8) The apparent ileal digestibility (AID) and standardized ileal digestibility (SID) of CP and AAs of corn (29):

$$\mathrm{AID}\;\left(\%\right)=\left[1-\left(\frac{AA_{\mathrm{digesta}}}{AA_{\mathrm{diet}}}\right)\times \left(\frac{Cr_{\mathrm{diet}}}{Cr_{\mathrm{digesta}}}\right)\right]\times 100\%$$

$$SID\;\left(\%\right)=\mathrm{AID}+\left(\frac{IAA_{\mathrm{end}}}{AA_{\mathrm{diet}}}\right)\times 100$$

$$IAA_{\mathrm{end}}\left(\%\right)=\left[AA_{\mathrm{digesta}}\times \left(\frac{Cr_{\mathrm{diet}}}{Cr_{\mathrm{digesta}}}\right)\right]$$

where AA_digesta_ is the AA concentrations in the ileal digesta (g/kg of DM); AA_diet_ is the AA concentrations in the diets (g/kg of DM); Cr_diet_ is the chromium concentration in the diet (g/kg of DM); Cr_digesta_ is the chromium concentration in the ileal digesta (g/kg of DM); IAA_end_ is the basal ileal endogenous loss of AAs (g/kg of DM intake).

### 2.2 Exp.2: The digestibility, emission, and deposition of minerals in two new corn varieties fed to broilers

The animal trial was conducted in the New Hope Liuhe Technology Research and Development Center in Pingdu (Shandong, China). One-hundred and twenty-six 10-day-old healthy Ross 308 broilers with similar initial body weight of 249.89 ± 1.47 g were selected and randomly divided into 3 treatments groups, with 6 replicated metabolic cages per treatment and 7 chickens per cage. The experiment diet containing 99.6% corn and 0.4% Cr_2_O_3_ as insert indicator. Due to high sensitivity of broilers to relative high levels of Cd, only the conventional corn, low-phytate phosphorus corn and high-iron corn were used in this trial. All diets were meshed and went through 2.5 mm sieve. The animal trial lasted for 15 days, including 8 days for diet and metabolic cages adaptation, and 7 days for total feces collection. Broilers had free access to feed and drink, and a constant light–dark cycle of L16D8 and a constant temperature of 26°C were kept in the room. In addition, conventional feeding operation procedures and routine immunization procedures were conducted.

The feces collected for 7 days were mixed and weighted, then dried at 65°C for 48 h and kept at room temperature for 24 h. Approximately 200 g of subsamples was taken and kept at −20°C for further analysis. The contents of DM, CP, GE, and mineral contents (e.g., Ca, P, Fe) in feces were determined followed the same procedure in *Exp. 1*. At the end of the animal trial, one chicken close to the average body weight of each treatment group from each replicate cage was chosen for slaughter. The liver and left tibia of each chicken was collected and kept at −20°C for further analysis. The length and width of the tibia were measured before storage. Moreover, the digesta in the ileum (with the last 4 cm discarded) was collected and kept at −20°C, and the Fe, P and Cr content in digesta were determined followed the same procedure in *Exp. 1*.

Approximately 1 ~ 5 g of liver sample and tibia sample (need to be degreased with petroleum ether before analysis) were dried at 130°C until completely carbonized, and then kept at 550 ± 20°C for ashing for 3 h and dissolved in hydrochloric acid for analysis. The Fe content in liver and tibia samples and the contents of Ca and P in tibia samples were determined following the same procedure in *Exp. 1*.

The major formula used for calculation in *Exp. 2* were the same as those in *Exp. 1* except for:

Ca or P deposition in tibia:

$$Ca\;\mathrm{or}\;P\;\mathrm{deposition}\;\mathrm{in}\;\mathrm{tibia}\;\left(\%\right)=2\times \left[\begin{array}{l} \mathrm{ash}\;\mathrm{weight}\;\mathrm{of}\;\mathrm{left}\;\mathrm{tibia}\left(g\right)\\ \times \;Ca\;\mathrm{or}\;P\;\mathrm{content}\;\mathrm{in}\;\mathrm{ash}\;\mathrm{of}\;\\ \mathrm{the}\;\mathrm{left}\;\mathrm{tibia}\left(\%\right) \end{array}\right]\times 100$$

### 2.3 Statistical analyses

All data were checked for normality and homogeneity of variance using the PROC UNIVERIATE procedure in SAS 9.4 (SAS Institute Inc., Carry, NC, USA), and outliers were detected and removed. One-way ANOVA was conducted using the PROC MIXED procedure in SAS 9.4. In Exp.1, the statistical model included the fixed effect of the dietary treatment and the random effects of the chamber and phase. In Exp.2, dietary treatment was the only fixed effect. The mean values for each treatment were calculated using the LSMEANS statement of SAS 9.4, and multiple comparisons were adjusted using Tukey’s test, with *p* < 0.05 as significant difference and 0.05 ≤ *p* < 0.10 as a trend of difference.

## 3 Results and discussion

### 3.1 Exp.1: chemical compositions, available energy, and amino acid digestibility in three new corn varieties fed to growing pigs

The chemical compositions of the 3 new corn varieties and the conventional corn are shown in Table 2. The starch content of the high-iron corn and the cadmium-resistant corn was numerically greater than that of the low-phytate phosphorus corn and the conventional corn, all slightly greater than previously reported values (30, 31), possibly due to differences in corn varieties or analytical methods used. The CP content in all 4 corn varieties was around 7%, all slightly lower than previously reported values (30, 32–34), which was in accordance with their increased starch content. The NDF, ADF and GE contents of the high-iron corn were numerically lower than those of the other 3 corn varieties. The iron content in the high-iron corn and the cadmium content in the cadmium-resistant corn were numerically greater than those in the other corns varieties, while the phosphorus content in the low-phytate phosphorus corn was numerically slightly greater than the other corn varieties, indicating the new varieties have achieved the designed goal of corn breeding. Except for cystine, methionine and tryptophan, all the other 15 amino acids contents were numerically greater in the high-iron corn than in the other three corn varieties, which may be attributed to the significance of iron as a cofactor for several functional proteins (35). In high-iron corn, the contents of calcium, copper, manganese, and zinc were all numerically greater than those in the conventional corn, exhibiting the interactive effects for mineral accumulation that could be influenced by genetic factors.

**Table 2:** Analyzed chemical compositions and amino acid contents of three new corn varieties and the conventional corn used in this study (% of dry matter, unless otherwise indicated).

Item	Conventional corn	High-iron corn	Cadmium-resistant corn	Low-phytate phosphorus corn
Proximate analysis, %				
Dry matter	89.00	89.69	89.52	89.56
Starch	67.68	69.06	68.95	67.35
Crude protein	7.06	7.05	7.02	7.00
Ether extract	3.36	3.28	3.26	3.32
Ash	1.37	1.36	1.31	1.35
Neutral detergent fiber	9.19	8.53	8.95	9.06
Acid detergent fiber	2.42	2.18	2.50	2.21
Gross energy, MJ/kg	16.91	16.75	16.89	16.90
Minerals, mg/kg				
Calcium	0.0151	<0.015	<0.015	0.0185
Total phosphorus	0.300	0.300	0.300	0.310
Iron	27.936	29.608	28.628	26.926
Copper	1.920	3.232	2.317	1.582
Manganese	5.111	5.208	4.993	4.874
Zinc	23.406	24.644	23.829	24.084
Cadmium	0.0018	0.0027	0.0057	0.0007
Amino acids, %				
Asp	0.42	0.47	0.43	0.41
Thr	0.23	0.26	0.24	0.23
Ser	0.30	0.33	0.29	0.29
Glu	1.10	1.20	1.10	1.06
Pro	0.49	0.60	0.54	0.50
Gly	0.28	0.32	0.29	0.27
Ala	0.44	0.51	0.46	0.44
Cys	0.16	0.16	0.15	0.15
Val	0.30	0.34	0.30	0.29
Met	0.16	0.17	0.18	0.15
Ile	0.21	0.23	0.21	0.20
Leu	0.72	0.78	0.70	0.69
Tyr	0.25	0.26	0.17	0.23
Phe	0.30	0.33	0.29	0.29
His	0.19	0.21	0.19	0.18
Lys	0.25	0.28	0.24	0.23
Arg	0.34	0.37	0.31	0.31
Trp	0.06	0.06	0.05	0.05

The ATTD of nutrients and available energy contents of the 3 new corn varieties and the conventional corn fed to growing pigs are shown in Table 3. The ATTD of NDF was significantly lower in the high-iron corn than that in the conventional corns (*p* = 0.015), while the ATTD of ADF was significantly lower in the high-iron corn and the low-phytate phosphorus corn than the other two corn varieties (*p* = 0.008), which may be due to the low NDF and ADF levels in the high-iron corn. In addition, the ATTD of GE and EE in the low-phytate phosphorus corn tended to be lower than those in the conventional corn (*p* = 0.066 and 0.092, respectively). There were no significant differences in ATTD of CP and organic matter (OM) of the 4 corn varieties in growing pigs, which was in accordance with the similar CP and ash contents in those 4 corn varieties.

**Table 3:** The apparent total tract digestibility (ATTD) of nutrients and available energy contents of the three new corn varieties and the conventional corn fed to growing pigs (as-fed basis).

Item	Conventional corn	High-iron corn	Cadmium-resistant corn	Low-phytate phosphorus corn	SEM	*p-*value
ATTD, %						
Gross energy	91.89	90.33	89.84	89.57	0.65	0.066
Crude protein	83.40	75.66	79.45	80.04	2.28	0.17
Ether extract	70.16	61.95	62.92	60.32	2.77	0.092
Neutral detergent fiber	67.21^a^	52.18^b^	56.67^ab^	56.00^ab^	3.04	0.015
Acid detergent fiber	68.36^a^	52.59^b^	61.51^ab^	55.33^b^	3.06	0.008
Organic matter	93.29	91.21	91.38	91.55	0.64	0.10
Available energy, MJ/kg						
Digestible energy	15.07^a^	14.49^b^	14.70^ab^	14.60^ab^	0.14	0.040
Metabolizable energy	14.53	13.98	14.09	14.12	0.18	0.19
Net energy	10.62	10.86	10.84	10.75	0.11	0.19

Means within a row with unlike superscript letters were significantly different (*p* < 0.05, *n* = 6).

In the current study, the DE, ME and NE values of the 4 corn varieties ranged from 14.49 MJ/kg to 15.07 MJ/kg, 13.98 MJ/kg to 14.53 MJ/kg, and 10.62 MJ/kg to 10.86 MJ/kg, respectively, comparable to the DE and ME values but lower than the NE value provided by NRC (2012) (3), which may be because the latter is a calculated value using prediction equation. Moreover, the DE value of the high iron corn in growing pigs were significantly lower than those of the conventional corn (*p* = 0.040), and there were no significant differences on the ME and NE values among the 4 corn varieties, indicating that the reference energy values may not be a particular concern when formulating a swine diet with the 4 different corn varieties, especially when using the ME or NE system.

The AID and SID of AAs of the 3 new corn varieties and the conventional corn fed to growing pigs are shown in Table 4. The AID of Tyr was significantly greater in the low-phytate phosphorus corn and the high-iron corn than that in the cadmium-resistant corn (*p* = 0.003), and the SID of Tyr was significantly greater in the low-phytate phosphorus corn than that in the cadmium-resistant corn (*p* = 0.023). For the other AAs, the AID and SID were similar among the 4 different corn varieties, indicating that the reference AAs contents may also not be a particular concern when formulating a swine diet with the 4 different corn varieties, especially when using the AID and SID system.

**Table 4:** The apparent ileal digestibility (AID) and standardized ileal digestibility (SID) of amino acids of the three new corn varieties and the conventional corn fed to growing pigs.

Item	Conventional corn	High-iron corn	Cadmium-resistant corn	Low-phytate phosphorus corn	SEM	*P-*value
AID, %						
Asp	49.97	57.26	56.81	60.56	2.81	0.12
Thr	34.28	43.36	41.96	45.98	3.70	0.22
Ser	56.39	59.04	59.36	63.35	2.98	0.47
Glu	71.54	76.06	77.01	77.22	1.65	0.12
Pro	48.05	53.60	49.50	40.42	5.68	0.43
Gly	37.69	33.29	37.04	33.96	5.39	0.92
Ala	57.70	63.79	66.57	68.67	3.04	0.12
Cys	65.17	66.65	69.79	69.75	2.68	0.55
Val	55.51	61.34	61.43	62.03	2.19	0.21
Met	72.77	75.28	79.67	77.33	2.08	0.17
Ile	55.91	60.98	60.89	63.85	2.06	0.11
Leu	73.20	76.06	76.21	77.91	1.64	0.31
Tyr	69.73^ab^	71.28^a^	61.61^b^	73.91^a^	2.02	0.003
Phe	68.41	72.32	72.84	74.25	1.55	0.12
His	66.61	69.68	70.41	70.10	2.03	0.60
Lys	49.58	52.31	49.07	54.53	3.17	0.55
Arg	70.72	69.70	72.06	73.55	3.88	0.90
Trp	49.11	50.16	44.64	48.20	4.08	0.77
SID, %						
Asp	64.36	70.18	70.86	75.21	2.81	0.11
Thr	48.85	56.35	56.26	60.62	3.70	0.23
Ser	67.07	68.76	70.40	74.46	2.98	0.37
Glu	79.02	82.87	84.44	84.98	1.65	0.11
Pro	58.69	62.29	59.29	50.95	5.68	0.54
Gly	54.21	48.04	53.47	51.14	5.39	0.84
Ala	66.16	71.17	74.67	77.13	3.04	0.12
Cys	73.42	75.00	78.78	78.68	2.68	0.43
Val	66.32	71.02	72.16	73.39	2.19	0.19
Met	77.15	79.53	83.52	82.09	2.08	0.20
Ile	67.37	71.39	72.51	75.71	2.06	0.085
Leu	79.38	81.77	82.55	84.35	1.63	0.25
Tyr	76.92^ab^	78.36^ab^	72.46^b^	81.98^a^	2.02	0.023
Phe	76.79	79.91	81.30	82.91	1.55	0.089
His	74.62	77.00	78.44	78.68	2.03	0.54
Lys	59.00	64.06	62.61	68.70	3.23	0.25
Arg	77.07	75.45	78.86	80.43	3.88	0.81
Trp	62.40	63.45	60.59	64.15	4.08	0.92

Means within a row with unlike superscript letters were significantly different (*p* < 0.05, *n* = 6).

### 3.2 The digestibility, emission, and deposition of minerals in two new corn varieties fed to broilers

In this animal trial, the average feed intake of all broilers was about 20 g, and the average daily gain ranged from 2 ~ 13 g, among which 10 cages showed negative growth (data not shown), which may be due to the unbalanced nutrition of the designed diets that only contained corn. The ATTD and AID of nutrients of 2 new corn varieties and the conventional corn fed to broilers are shown in Table 5. The ATTD of Fe, Cu, Mn, and Zn were all negative, and the AID of iron in each group was also negative (data not shown), which may be due to the endogenous secretion in the upper intestine. There is no significant difference in the ATTD of DM, GE, CP, and phosphorus among the 3 treatment groups, and there is also no significant difference in the AID of phosphorus among the 3 treatment groups. Based on the ATTD of GE and the determined GE values in the corn, the apparent metabolizable energy (AME) values of the conventional corn, the low-phytate phosphorus corn and the high-iron corn on broilers were 12.94 MJ/kg, 12.92 MJ/kg and 12.85 MJ/kg, respectively, which had no significant differences. However, those determined AME values were all lower than the values of 14.016 MJ/kg reported in NRC (1994) (36), which may be due to the differences in chicken breeds used.

**Table 5:** The apparent total tract digestibility (ATTD) and apparent ileal digestibility (AID) of nutrients of two new corn varieties and the conventional corn fed to broilers.

Item	Conventional corn	Low-phytate phosphorus corn	High-iron corn	SEM	*P*-value
ATTD, %					
Dry matter	74.41	74.89	74.51	0.488	0.460
Gross Energy	76.51	76.44	76.71	0.453	0.817
Crude protein	29.93	31.94	32.13	2.097	0.903
Phosphorus	16.63	13.89	13.28	1.348	0.573
AID, %					
Phosphorus	53.71	56.31	70.87	5.079	0.349

*n* = 6.

The emission of minerals in feces of broilers fed 2 new corn varieties and the conventional corn is shown in Table 6. The iron emission in feces of broilers fed the low-phytate phosphorus corn was significantly lower than those fed the conventional corn and the high-iron corn (*p* = 0.023), which may be related to the fact that the iron content in the low-phytate phosphorus corn was lower than that in the other two groups. Moreover, broilers fed the high-iron corn showed significantly greater manganese (Mn) emission in feces compared with those fed the low-phytate phosphorus corn (*p* = 0.01) and significantly greater plumbum (Pb) emission in feces compared with those fed the conventional corn (*p* = 0.02), and tended to show greater Ca emission in feces compared with the other two groups (*p* = 0.07), indicating that utilization of the new high-iron corn in broiler feed may cause some environment problems considering the mineral emission in feces.

**Table 6:** The emission of minerals in feces of broilers fed 2 new corn varieties and the conventional corn.

Item	Conventional corn	Low-phytate phosphorus corn	High-iron corn	SEM	*p*-value
Calcium, %	0.26	0.28	0.31	0.01	0.07
Phosphorus, %	0.99	1.05	1.04	0.02	0.39
Copper, mg/kg	7.58	7.57	7.68	0.23	0.98
Iron, mg/kg	171.67^a^	102.83^b^	181.67^a^	10.33	0.001
Manganese, mg/kg	18.17^ab^	16.50^b^	19.83^a^	0.50	0.01
Zinc, mg/kg	133.33	141.67	130.00	4.59	0.59
Plumbum, mg/kg	0.26^b^	0.38^ab^	0.50^a^	0.04	0.02
Arsenic, mg/kg	0.14	0.13	0.15	0.01	0.41

Means within a row with unlike superscript letters were significantly different (*p* < 0.05, *n* = 6).

The tibia development and deposition of minerals in tibia and liver of broilers fed 2 new corn varieties and the conventional corn is shown in Table 7. The tibial length of broilers fed the low-phytate phosphorus corn and the high-iron corn is significantly lower than those fed the conventional corn (*p* = 0.01), but the tibial width was not affected. There was no significant difference in the levels of iron deposition in tibia and liver, as well as the levels of calcium and phosphorus deposition in tibia. Lack of calcium and phosphorus in the diet or an unreasonable ratio of calcium and phosphorus will lead to poor bone development in broilers. In broilers aged 1–21 days, phosphorus deficiency-related skeletal dysplasia is characterized by widening of the growth plate at the end of the bone epiphysis (37). In the current study, the reduced tibia length of broilers in the low-phytate phosphorus corn group may indicate that the low-phytate phosphorus corn did not achieve the breeding goal to some extent.

**Table 7:** The tibia development and deposition of minerals in tibia and liver of broilers fed two new corn varieties and the conventional corn.

Item	Conventional corn	Low-phytate phosphorus corn	High-iron corn	SEM	*P*-value
Tibia development, mm					
Tibial length	63.57^a^	61.35^b^	60.91^b^	0.506	0.010
Tibial width	4.09	4.06	4.12	0.058	0.930
Mineral deposition in tibia, mg/kg					
Iron	98.33	86.67	81.33	4.032	0.221
Calcium	57.72	58.24	61.96	1.380	0.418
Phosphorus	31.87	29.64	30.18	0.836	0.552
Mineral deposition in liver, mg/kg					
Iron	98.33	86.67	81.33	4.032	0.221

Means within a row with unlike superscript letters were significantly different (*p* < 0.05, *n* = 6).

## 4 Conclusion

The chemical compositions varied among the 3 new corn varieties developed through modern breeding techniques. However, those breeding techniques rarely changed the nutrient digestibility and the available energy values of the corn when fed to growing pigs and broilers. Those techniques could provide new plant ingredients that which have potential benefits to pig and broiler production, but the comprehensive effects may be better when applied to growing pigs considering both the growth performance and the environmental effects. However, additional animal trials with larger populations and more balanced diets are needed to further explore the utilization of those new corn varieties in animal feed.

## Data availability statement

The original contributions presented in the study are included in the article/Supplementary material, further inquiries can be directed to the corresponding authors.

## Ethics statement

The animal studies were approved by Institutional Animal Care and Use Committee of China Agricultural University. The studies were conducted in accordance with the local legislation and institutional requirements. Written informed consent was obtained from the owners for the participation of their animals in this study.

## Author contributions

HC: Methodology, Writing – original draft, Writing – review & editing. LY: Formal analysis, Investigation, Writing – review & editing. XS: Investigation, Writing – review & editing. LL: Investigation, Writing – review & editing. SX: Project administration, Writing – review & editing. SH: Project administration, Supervision, Writing – review & editing. SZ: Conceptualization, Project administration, Supervision, Writing – review & editing.
