# Supplementary material for: Determining the nutritional values of new corn varieties on pigs and broilers
Source: Front Vet Sci. 2024 Feb 8;11:1358773. doi: 10.3389/fvets.2024.1358773 (PMC10881873; doi:10.3389/fvets.2024.1358773)
Supplement: Supplementary file 1 [file Data_Sheet_1.docx]

Supplementary Material

# Supplementary Tables

**Table 1.** Analyzed chemical compositions and amino acid contents of three new corn varieties and the conventional corn used in this study (% of dry matter, unless otherwise indicated).

| Item | conventional corn | high-iron corn | cadmium-resistant corn | low-phytate phosphorus corn |
| --- | --- | --- | --- | --- |
| Proximate analysis，% |  |  |  |  |
| Dry matter | 89.00 | 89.69 | 89.52 | 89.56 |
| Starch | 67.68 | 69.06 | 68.95 | 67.35 |
| Crude protein | 7.06 | 7.05 | 7.02 | 7.00 |
| Ether extract | 3.36 | 3.28 | 3.26 | 3.32 |
| Ash | 1.37 | 1.36 | 1.31 | 1.35 |
| Neutral detergent fiber | 9.19 | 8.53 | 8.95 | 9.06 |
| Acid detergent fiber | 2.42 | 2.18 | 2.50 | 2.21 |
| Gross energy, MJ/kg | 16.91 | 16.75 | 16.89 | 16.90 |
| Minerals, mg/kg |  |  |  |  |
| Calcium | 0.0151 | <0.015 | <0.015 | 0.0185 |
| Total phosphorus | 0.300 | 0.300 | 0.300 | 0.310 |
| Iron | 27.936 | 29.608 | 28.628 | 26.926 |
| Copper | 1.920 | 3.232 | 2.317 | 1.582 |
| Manganese | 5.111 | 5.208 | 4.993 | 4.874 |
| Zinc | 23.406 | 24.644 | 23.829 | 24.084 |
| Cadmium | 0.0018 | 0.0027 | 0.0057 | 0.0007 |
| Amino acids, % |  |  |  |  |
| Asp | 0.42 | 0.47 | 0.43 | 0.41 |
| Thr | 0.23 | 0.26 | 0.24 | 0.23 |
| Ser | 0.30 | 0.33 | 0.29 | 0.29 |
| Glu | 1.10 | 1.20 | 1.10 | 1.06 |
| Pro | 0.49 | 0.60 | 0.54 | 0.50 |
| Gly | 0.28 | 0.32 | 0.29 | 0.27 |
| Ala | 0.44 | 0.51 | 0.46 | 0.44 |
| Cys | 0.16 | 0.16 | 0.15 | 0.15 |
| Val | 0.30 | 0.34 | 0.30 | 0.29 |
| Met | 0.16 | 0.17 | 0.18 | 0.15 |
| Ile | 0.21 | 0.23 | 0.21 | 0.20 |
| Leu | 0.72 | 0.78 | 0.70 | 0.69 |
| Tyr | 0.25 | 0.26 | 0.17 | 0.23 |
| Phe | 0.30 | 0.33 | 0.29 | 0.29 |
| His | 0.19 | 0.21 | 0.19 | 0.18 |
| Lys | 0.25 | 0.28 | 0.24 | 0.23 |
| Arg | 0.34 | 0.37 | 0.31 | 0.31 |
| Trp | 0.06 | 0.06 | 0.05 | 0.05 |

**Table 2.** The apparent ileal digestibility (AID) and standardized ileal digestibility (SID) of amino acids of the three new corn varieties and the conventional corn fed to growing pigs.

| Item | conventional corn | high-iron corn | cadmium-resistant corn | low-phytate phosphorus corn | SEM | *P-*value |
| --- | --- | --- | --- | --- | --- | --- |
| AID,% |  |  |  |  |  |  |
| Asp | 49.97 | 57.26 | 56.81 | 60.56 | 2.81 | 0.12 |
| Thr | 34.28 | 43.36 | 41.96 | 45.98 | 3.70 | 0.22 |
| Ser | 56.39 | 59.04 | 59.36 | 63.35 | 2.98 | 0.47 |
| Glu | 71.54 | 76.06 | 77.01 | 77.22 | 1.65 | 0.12 |
| Pro | 48.05 | 53.60 | 49.50 | 40.42 | 5.68 | 0.43 |
| Gly | 37.69 | 33.29 | 37.04 | 33.96 | 5.39 | 0.92 |
| Ala | 57.70 | 63.79 | 66.57 | 68.67 | 3.04 | 0.12 |
| Cys | 65.17 | 66.65 | 69.79 | 69.75 | 2.68 | 0.55 |
| Val | 55.51 | 61.34 | 61.43 | 62.03 | 2.19 | 0.21 |
| Met | 72.77 | 75.28 | 79.67 | 77.33 | 2.08 | 0.17 |
| Ile | 55.91 | 60.98 | 60.89 | 63.85 | 2.06 | 0.11 |
| Leu | 73.20 | 76.06 | 76.21 | 77.91 | 1.64 | 0.31 |
| Tyr | 69.73^ab^ | 71.28^a^ | 61.61^b^ | 73.91^a^ | 2.02 | 0.003 |
| Phe | 68.41 | 72.32 | 72.84 | 74.25 | 1.55 | 0.12 |
| His | 66.61 | 69.68 | 70.41 | 70.10 | 2.03 | 0.60 |
| Lys | 49.58 | 52.31 | 49.07 | 54.53 | 3.17 | 0.55 |
| Arg | 70.72 | 69.70 | 72.06 | 73.55 | 3.88 | 0.90 |
| Trp | 49.11 | 50.16 | 44.64 | 48.20 | 4.08 | 0.77 |
| SID, % |  |  |  |  |  |  |
| Asp | 64.36 | 70.18 | 70.86 | 75.21 | 2.81 | 0.11 |
| Thr | 48.85 | 56.35 | 56.26 | 60.62 | 3.70 | 0.23 |
| Ser | 67.07 | 68.76 | 70.40 | 74.46 | 2.98 | 0.37 |
| Glu | 79.02 | 82.87 | 84.44 | 84.98 | 1.65 | 0.11 |
| Pro | 58.69 | 62.29 | 59.29 | 50.95 | 5.68 | 0.54 |
| Gly | 54.21 | 48.04 | 53.47 | 51.14 | 5.39 | 0.84 |
| Ala | 66.16 | 71.17 | 74.67 | 77.13 | 3.04 | 0.12 |
| Cys | 73.42 | 75.00 | 78.78 | 78.68 | 2.68 | 0.43 |
| Val | 66.32 | 71.02 | 72.16 | 73.39 | 2.19 | 0.19 |
| Met | 77.15 | 79.53 | 83.52 | 82.09 | 2.08 | 0.20 |
| Ile | 67.37 | 71.39 | 72.51 | 75.71 | 2.06 | 0.085 |
| Leu | 79.38 | 81.77 | 82.55 | 84.35 | 1.63 | 0.25 |
| Tyr | 76.92^ab^ | 78.36^ab^ | 72.46^b^ | 81.98^a^ | 2.02 | 0.023 |
| Phe | 76.79 | 79.91 | 81.30 | 82.91 | 1.55 | 0.089 |
| His | 74.62 | 77.00 | 78.44 | 78.68 | 2.03 | 0.54 |
| Lys | 59.00 | 64.06 | 62.61 | 68.70 | 3.23 | 0.25 |
| Arg | 77.07 | 75.45 | 78.86 | 80.43 | 3.88 | 0.81 |
| Trp | 62.40 | 63.45 | 60.59 | 64.15 | 4.08 | 0.92 |
